# Comorbidities as a Personalized Target in Patients with Severe Asthma Treated with Dupilumab

**DOI:** 10.3390/jpm15100471

**Published:** 2025-10-02

**Authors:** Carlota Gonzalez-Lluch, Maria Basagaña, Laura Pardo, Paula Cruz Toro, Agnes Hernandez-Biette, Carlos Martinez-Rivera, Ignasi Garcia-Olive

**Affiliations:** 1 Department of Ear, Nose and Throat, Hospital Universitari Germans Trias i Pujol, 08916 Badalona, Barcelona, Spainpcruzt.germanstrias@gencat.cat (P.C.T.); 2 Department of Allergology, Hospital Universitari Germans Trias i Pujol, 08916 Badalona, Barcelona, Spain; mbasagana.germanstrias@gencat.cat; 3 Department of Respiratory Medicine, Hospital Universitari Germans Trias i Pujol, 08916 Badalona, Barcelona, Spaincmartinez.germanstrias@gencat.cat (C.M.-R.); 4 Institut de Recerca Germans Trias i Pujol, 08916 Badalona, Barcelona, Spain; 5 Departament de Medicina, Universitat Autonònoma de Barcelona, 08916 Bellaterra, Barcelona, Spain

**Keywords:** asthma, comorbidities, dupilumab, personalized medicine, real-life study

## Abstract

**Background:** This study aimed to evaluate the clinical effectiveness of dupilumab and its impact on CRSwNP and type 2 inflammatory biomarkers in patients with severe uncontrolled asthma, with or without comorbidities, within a real-life cohort. **Methods:** This was a single-center, prospective, and observational real-life study conducted at the Severe Asthma Unit of Germans Trias i Pujol University Hospital. The objective of this study was to assess the real-world response to dupilumab treatment in patients with severe asthma, with or without nasal polyposis, bronchiectasis, obesity, or switching from another biologic drug for their asthma. **Results:** The ACT score significantly increased (13.7 vs. 20.6; *p* = 0.001), while the number of exacerbations decreased (3.1 vs. 0.7; *p* = 0.005). Patients with CRSwNP showed an increase in the ACT score (13.1 vs. 19.8; *p* = 0.011) and a decrease in the number of exacerbations (3.0 vs. 1.3; *p* = 0.217). Patients with nasal polyps showed an increase in both SNOT22 (78.3 vs. 38.3; *p* = 0.013) and global VAS (8 vs. 4.2; *p* = 0.028). Patients with bronchiectasis receiving dupilumab showed an increase in the ACT score (12.7 vs. 21.3; *p* = 0.039) and a marked decrease in the number of exacerbations (2.8 vs. 0; *p* = 0.025). Obese patients treated with dupilumab showed an improvement in the ACT score (14.1 vs. 21.3; *p* = 0.044) and a decrease in the rate of exacerbations (3.2 vs. 1.3; *p* = 0.030). Patients with rhinoconjunctivitis receiving dupilumab showed an increase in the ACT score (13.4 vs. 19.1; *p* = 0.017) and a decrease in the number of exacerbations (3.3 vs. 0.8; *p* = 0.024). **Conclusions:** Dupilumab is a highly effective treatment for severe asthma, showing significant improvements in lung function, reductions in exacerbations, and enhanced quality of life for patients with and without nasal polyps. The results of this real-life study support dupilumab as a valuable addition to the therapeutic armamentarium for asthma, particularly for those with type 2 inflammation despite the presence of comorbidities such as bronchiectasis or obesity, or for patients in whom a previous biologic treatment failed.

## 1. Introduction

Severe uncontrolled asthma represents a multifactorial and challenging clinical entity that continues to impose a significant burden on patients and healthcare systems alike. Although it accounts for only approximately 5–10% of the total asthma population [1], its impact is disproportionately large due to frequent exacerbations, hospitalizations, and reduced quality of life. Within this subset, a substantial proportion—estimated at 70–80%—present with a type 2 inflammation endotype, which is biologically characterized by elevated eosinophil counts, increased fractional exhaled nitric oxide (FeNO), and heightened serum immunoglobulin E (IgE) levels [2]. These biomarkers reflect the underlying immunopathology driven by cytokines such as interleukin-4 (IL-4), interleukin-5 (IL-5), and interleukin-13 (IL-13), which are key therapeutic targets in modern asthma management. Several biologic drugs are available to treat patients with severe asthma, highlighting the need for personalized strategies based on laboratory tests and comorbidity burden.

The advent of biologic therapies has dramatically reshaped the treatment landscape for severe asthma, particularly in patients with type 2 inflammation who remain symptomatic despite high-dose inhaled corticosteroids and additional controller medications. Among these biologics, dupilumab has emerged as a highly promising agent. It is a fully human monoclonal antibody that specifically blocks the shared receptor component for IL-4 and IL-13, thereby inhibiting downstream signaling pathways involved in type 2 inflammatory responses. Clinical trials and real-world studies have consistently demonstrated the efficacy and safety of dupilumab, showing significant reductions in asthma exacerbations, improvements in lung function parameters (such as FEV_1_), and enhanced symptom control in patients with elevated type 2 biomarkers [3,4].

Nonetheless, the management of severe asthma frequently extends beyond the control of airway inflammation alone. A wide array of comorbidities commonly coexist in these patients, further complicating clinical care and adversely affecting the overall disease burden [5]. These comorbidities can be categorized as potentially T2-related comorbidities, potentially oral corticosteroid (OCS)-related comorbidities, and comorbidities that mimic or aggravate asthma [5]. Conditions such as chronic rhinosinusitis with nasal polyps (CRSwNP), bronchiectasis, and atopic dermatitis are prevalent in this population and are often associated with persistent inflammation and poorer health-related quality of life [3]. These comorbid conditions, many of which also share a type 2 inflammatory basis, may influence a treatment response and need to be considered when tailoring biologic therapies, especially when these comorbidities increase in number [5] or are measured with the multimorbidity in difficult asthma score (MiDAS) [6].

Dupilumab, beyond its indication in asthma, has also been approved for the treatment of moderate-to-severe atopic dermatitis and has demonstrated clinical benefit in patients with CRSwNP, making it an attractive option in cases where these comorbidities coexist. Its broad efficacy across type 2-driven diseases is supported by randomized controlled trials that show significant improvement in both upper and lower airway symptoms, reduced polyp size, and enhanced quality-of-life measures [1,7].

Given this background, this study was designed to evaluate the real-world clinical effectiveness of dupilumab in a cohort of patients with severe uncontrolled asthma, with a particular focus on its impact on those with and without relevant comorbidities. Special attention was directed to its effects on CRSwNP, as well as on type 2 inflammatory biomarkers such as eosinophil count, FeNO, and IgE levels. This real-life evidence is crucial to complement findings from clinical trials and to better understand dupilumab’s performance in a more heterogeneous and comorbidity-burdened population.

Our aim was to analyze the impacts of comorbidities on the clinical response to dupilumab in patients with severe asthma and the drug response in patients previously treated with another biologic for asthma.

## 2. Methods

This investigation was designed as a single-center, prospective, observational, real-life study, conducted at the Severe Asthma Unit of the Germans Trias i Pujol University Hospital. This study’s primary objective was to comprehensively evaluate the real-world clinical response to dupilumab treatment in a heterogeneous population of patients diagnosed with severe uncontrolled asthma. This population included individuals with either potentially T2-related comorbidities, such as nasal polyposis or eosinophilic esophagitis; potentially OCS-related comorbidities, such as obesity; or comorbidities that could mimic asthma, such as bronchiectasis, as well as patients who transitioned to dupilumab after prior treatment with other biologic agents targeting asthma. This study aimed to reflect the complexities and variations encountered in everyday clinical practice by encompassing these diverse patient subgroups.

### 2.1. Setting

This study was carried out at the Germans Trias i Pujol University Hospital, a tertiary care center located in Badalona (Barcelona, Spain), which is the reference health center for the Northern Metropolitan Area in Barcelona. The hospital houses a specialized Severe Asthma Unit, staffed by a multidisciplinary team comprising pulmonologists, allergists, pediatricians, otolaryngologists, and pharmacists. This team collaborates closely to ensure a holistic and individualized approach in managing patients with uncontrolled severe asthma. The unit utilizes a systematic evaluation process that incorporates detailed phenotyping of asthma and an assessment of coexisting comorbidities to guide the selection of the most appropriate biological therapy. Monthly multidisciplinary committee meetings are held to discuss each patient’s case and, once a treatment plan is agreed upon, patients can typically initiate biologic therapy within one week at the hospital’s respiratory day unit. This streamlined pathway facilitates the timely initiation of biologic therapy, aiming to improve patient outcomes and adherence.

### 2.2. Population

This study prospectively enrolled all adult patients who initiated treatment with dupilumab for severe uncontrolled asthma between December 2021 and June 2023. The inclusion criteria focused on patients with asthma that remained uncontrolled despite standard high-intensity therapies, encompassing those with additional respiratory or systemic comorbidities such as nasal polyposis, bronchiectasis, or obesity. Patients who had previously received other biologic treatments for asthma but were switched to dupilumab due to suboptimal responses or adverse effects were also included. This study adopted a real-life observational design, capturing routine clinical practice data without interference in standard care pathways. The only exclusion criterion applied was the refusal or inability to provide informed consent, ensuring ethical compliance while maximizing external validity.

### 2.3. Statistical Analysis

Paired-sample *t*-tests were used to assess the impact of dupilumab treatment on clinical and inflammatory parameters. These tests evaluated whether the mean difference between baseline and post-treatment measurements in key variables—including the asthma control test (ACT) score, forced expiratory volume in one second (FEV_1_), serum IgE levels, blood eosinophil counts, and the number of asthma exacerbations—was statistically significantly different from zero. Analyses were conducted for the overall study cohort and stratified into various clinically relevant subgroups, including patients with and without chronic rhinosinusitis with nasal polyps (CRSwNP), those with and without bronchiectasis, obese versus non-obese patients, and individuals who initiated dupilumab following a switch from another biologic therapy. This subgroup analysis allowed for a nuanced understanding of treatment effectiveness across diverse clinical phenotypes and comorbidity profiles.

### 2.4. Ethical Aspects

The study protocol received approval from the regional ethics committee, specifically the Ethics Committee for Clinical Research of the Hospital Germans Trias i Pujol, approved on 15 January 2019, with Code PI-18-256. All procedures complied with the ethical standards laid out in the Declaration of Helsinki. Patients provided written informed consent prior to inclusion, ensuring respect for autonomy and confidentiality throughout this study.

## 3. Results

In total, 28 patients were included in the analysis. Table 1 lists the baseline characteristics of the 28 patients included in the analysis. The cohort was predominantly female (71.4%), with a mean age of 39.9 years and an average body mass index (BMI) of 27.8 kg/m^2^. Among them, 18 patients had allergic rhinoconjuntivitis (64.3%), 8 (28.6%) had a confirmed diagnosis of CRSwNP, 8 (28.6%) were obese, 7 patients (25.0%) had bronchiectasis, and 15 patients (53.6%) had undergone previous treatment with a biologic other than dupilumab (5 with mepolizumab, 4 with omalizumab, 4 with benralizumab, and 2 with reslizumab).

The clinical outcomes following dupilumab initiation in the 28 patients are summarized in Table 2. While there were no differences in pulmonary function, there was a significant increase in the ACT score from 13.7 at baseline to 10.6 at 12 months (*p* = 0.001), and a decrease in the number of exacerbations from 3.1 to 0.7 at 12 months (*p* = 0.005). The effect of dupilumab on asthma control in patients with severe asthma and nasal polyps is represented in Table 3. Patients with CRSwNP showed a significant increase in the ACT score (13.1 vs. 19.8; *p* = 0.011), while the decrease in the number of exacerbations (from 3.0 to 1.3) did not reach statistical significance (*p* = 0.217). Only 20 patients had a thoracic CT scan. Of these 20 patients, 7 (35.0%) had bronchiectasis. Patients with bronchiectasis receiving dupilumab showed an increase in the ACT score from 12.7 at baseline to 21.3 at 12 months (*p* = 0.039) and a marked decrease in the number of exacerbations (2.8 vs. 0; *p* = 0.025) (Table 4). The effect of dupilumab in obese patients is shown in Table 5. Obese patients treated with dupilumab showed an improvement in the ACT score (14.1 vs. 21.3; *p* = 0.044) and a decrease in the rate of exacerbations (3.2 vs. 1.3; *p* = 0.030) (Table 5). The number of exacerbations decreased from 3.8 at baseline to 1 at 12 months (*p* = 0.015) in patients who started treatment with dupilumab after switching from another therapy. There were no differences in the other variables analyzed (Table 6). Table 7 shows the results of dupilumab according to the presence of allergic rhinoconjunctivitis. Patients with rhinoconjunctivitis receiving dupilumab showed an increase in the ACT score (13.4 vs. 19.1; *p* = 0.017) and a decrease in the number of exacerbations (3.3 vs. 0.8; *p* = 0.024). No statistical significance was obtained in the analysis for comorbidities such as atopic dermatitis, food allergy, aspirin-exacerbated respiratory disease (AERD), or eosinophilic esophagitis due to the low prevalence of such conditions.

The results of dupilumab on nasal symptoms are shown in Table 8. Patients with nasal polyps showed an increase in both SNOT22 (78.3 vs. 38.3; *p* = 0.013) and global VAS (8 vs. 4.2; *p* = 0.028). Table 1 also shows the effect of dupilumab on asthma control in patients with severe asthma with or without bronchiectasis.

## 4. Discussion

This study provides further evidence supporting the efficacy of dupilumab in patients with severe asthma and comorbid chronic rhinosinusitis with nasal polyps (CRSwNP), reaffirming its role as a targeted therapy with dual benefits in the upper and lower airways. Dupilumab’s mechanism of action, which involves inhibition of the interleukin-4 receptor alpha subunit and blockade of both IL-4 and IL-13 signaling pathways, allows it to address type 2 inflammation comprehensively. This is particularly relevant in patients presenting with overlapping inflammatory diseases, such as asthma and CRSwNP, where shared immunopathological mechanisms are implicated. The dual benefit has been well documented in previous clinical trials and real-life studies, notably by Laidlaw et al. [8], who reported significant improvements in nasal congestion, sense of smell, and overall sinonasal-related quality of life. Our findings are consistent with these observations.

In our cohort, patients exhibited a notable reduction in sinonasal symptom burden, evidenced by a mean decrease of 40 points in the SNOT-22 score from baseline, along with a significant improvement in the global symptoms visual analog scale (VAS), which decreased from 8.0 to 4.2 after 12 months of dupilumab therapy. These results align closely with those of Förster-Ruhrmann et al. [9] and Mümmler et al. [10], who also demonstrated the superiority of dupilumab over other biologics in improving health-related quality of life in patients with CRSwNP. Improvements in olfactory function were similarly encouraging, as assessed by both the olfactory VAS and the Sniffin’ sticks TDI (threshold, discrimination, identification) test. This reinforces earlier findings from real-world settings and clinical trials [4,11,12] that have positioned dupilumab as the biologic with the most consistent and favorable impact on olfactory dysfunction among patients with CRSwNP.

Beyond sinonasal outcomes, our data demonstrated an overall clinical benefit in asthma control. Patients with CRSwNP experienced an increase in ACT scores and a reduction in annualized exacerbation rates. These findings support the rationale for treating severe asthma in the context of comorbid CRSwNP as a unified airway disease, in line with a precision medicine approach that recognizes the interconnectedness of upper and lower airway inflammation.

Importantly, when evaluating comorbidities beyond CRSwNP, we observed consistent improvements with dupilumab treatment. Patients with obesity showed significant clinical gains, including increased ACT scores and fewer exacerbations. These findings corroborate a prior meta-analysis by our group [13] and are aligned with preliminary results from a randomized study indicating that dupilumab improves pulmonary function and reduces exacerbation frequency regardless of body mass index [14]. However, contrasting data from Habenbacher et al. [15] suggest that obesity may attenuate the therapeutic response to dupilumab in CRSwNP specifically, highlighting the importance of patient stratification based on phenotype and comorbidity profile.

In addition, patients with allergic rhinoconjunctivitis in our sample demonstrated clinical improvement, echoing findings from earlier studies [16]. While the prevalence of other type 2 comorbidities, such as eosinophilic esophagitis [17] and atopic dermatitis [18], was low in our cohort—thus precluding statistical analysis—it is worth noting that dupilumab is approved for these indications, and the presence of such comorbidities may further support its selection in a personalized treatment algorithm.

The concept of personalized or precision medicine is especially relevant in severe asthma, where treatment decisions should be informed by the patient’s clinical phenotype, biomarker profile (e.g., blood eosinophil count and FeNO levels), and comorbidity burden. Dupilumab’s efficacy across multiple type 2 inflammatory diseases makes it an ideal candidate in patients with overlapping conditions, and our findings reinforce this principle. The holistic assessment of each patient’s inflammatory signature and disease expression allows for a tailored therapeutic approach, which improves outcomes and optimizes resource allocation in real-life clinical practice.

With regard to treatment switching, patients who transitioned to dupilumab after suboptimal responses to other biologics experienced a reduction in annualized exacerbations. Although most published data on switching to dupilumab have focused on improvements in sinonasal symptoms in patients with asthma and CRSwNP [19,20], our findings suggest that asthma control can also improve following such a switch. This is supported by recent evidence indicating that dupilumab provides comparable, if not superior, improvements in asthma control test (ACT) scores compared with intraclass switching following inadequate response to anti-IL-5 or anti-IL-5R monoclonal antibodies [21]. Dupilumab has also been described to reduce severe exacerbations and OCS use in patients with severe asthma in real-world settings [22]. These observations highlight the need to reassess biologic therapy periodically, particularly in patients with evolving clinical presentations or those with multiple comorbidities, and to consider dupilumab as a viable second-line option in the personalized care pathway.

Nonetheless, our study has limitations. As patients were recruited exclusively from a severe asthma unit, CRSwNP was typically a secondary indication for initiating dupilumab. This introduced a potential selection bias and may explain the relatively low mean baseline nasal polyp score (NPS) of 2.1, which was notably lower than values reported in other cohorts; for example, in the study of Fadda et al. [23], the mean NPS was 5.3. Despite this, we observed a mean NPS reduction of approximately 2 points, which was comparable in magnitude to other real-world and clinical trial findings, suggesting a consistent treatment effect even in populations with less severe nasal disease at baseline. Another limitation was the small sample size (especially within subgroups), which implies that some non-significant findings could be secondary to a limited statistical power.

In conclusion, our findings support the growing body of evidence indicating that dupilumab is an effective and well-tolerated therapeutic option for patients with severe asthma, particularly those with coexisting type 2 inflammatory comorbidities. Dupilumab is also a useful option in patients with previous treatment with other biologic drugs. The integration of personalized medicine principles—such as identifying patient-specific phenotypes, endotypes, and comorbidities—can enhance clinical decision making and optimize treatment outcomes. Further research with larger and more diverse populations, including stratification by biomarker status and comorbidity clustering, will be essential to fully harness the potential of dupilumab within individualized care models.

## Figures and Tables

**Table 1:** Baseline characteristics of patients included in this study.

Characteristics	*n* = 28
Age (years), m (SD)	39.9 (17.4)
Female, n (%)	20 (71.4)
BMI, m (SD)	27.8 (5.7)
Childhood-onset asthma, n (%)	18 (64.3)
Allergy, n (%)	22 (78.6)
Sensitized, n (%)	13 (86.7)
CRScNP, n (%)	8 (28.6)
AERD, n (%)	3 (10.7)
OCSs, n (%)	4 (14.3)
Inhaled steroid equivalent dose (mg), m (SD)	1120 (337)
Previous biologic treatment, n (%)	15 (53.6)
Previous CENS, n (%)	7 (25.0)
Thoracic CT scan, n (%)	20 (71.4)
Bronchiectasis	7(35.0)
Without bronchiectasis	13 (65.0)
Obesity, n (%)	8 (28.6)
Atopic dermatitis, n (%)	5 (17.9)
Allergic rhinoconjuntivitis, n (%)	18 (64.3)
Food allergy, n (%)	3.1 (10.7)
Eosinophilic esophagitis, n (%)	1 (3.6)
ACT, m (SD)	13.7 (5.4)
FEV1, m (SD)	73.7 (18.9)
FeNO, m (SD)	63.5 (51.1)
IgE, m (SD)	1056.8 (2330.2)
Eosinophil count, m (SD)	389.6 (459.1)
Comorbidities, n (%)	
None	8 (28.6)
One	6 (21.4)
Two or more	14 (50.0)

ACT: asthma control test; AERD: aspirin-exacerbated respiratory disease; ARC: allergic rhinoconjuntivitis; BMI: body mass index; CRSwNP: chronic rhinosinusitis with nasal polyps; FeNO: fractional exhaled nitric oxide; FEV1: forced expiratory volume in the first second; IgE: immunoglobulin; m: mean; n: number; OCSs: oral corticosteroids; SD: standard deviation.

**Table 2:** Impact of dupilumab in overall study population after 12 months of treatment.

	Global (*n* = 28)		
	Basal	12 Months	*p*-Value
ACT, m (SD)	13.7 (5.4)	20.6 (4.5)	0.001
FEV1, m (SD)	73.7 (18.9)	73.1 (18.9)	0.939
FeNO, m (SD)	63.5 (51.1)	29.8 (28.4)	0.085
IgE, m (SD)	1056.8 (2330.2)	681.8 (724.1)	0.702
Eosinophil count, m (SD)	389.6 (459.1)	624.4 (723.4)	0.256
AER, m (SD)	3.1 (2.9)	0.7 (1.5)	0.005

ACT: asthma control test; AER: annual exacerbation rate; ARC: allergic rhinoconjunctivitis; FeNO: fractional exhaled nitric oxide; FEV1: forced expiratory volume in the first second; IgE: immunoglobulin; SD: standard deviation.

**Table 3:** Impact of dupilumab according to the presence of nasal polyps after 12 months of treatment.

	Without CRScNP (*n* = 20)			With CRScNP (*n* = 8)		
	Basal	12 Months	*p*-Value	Basal	12 Months	*p*-Value
ACT, m (SD)	13.9 (6.1)	21.4 (4.9)	0.205	13.1 (3.3)	19.8 (4.5)	0.011
FEV1, m (SD)	78.1 (15.9)	74.7 (23.1)	0.723	63.2 (22.2)	71.5 (17.0)	0.531
FeNO, m (SD)	58.2 (49.7)	20.5 (8.5)	0.151	76.6 (55.7)	39.2 (39.6)	0.263
IgE, m (SD)	687.2 (646.6)	704.5 (164.7)	0.971	1980.2 (4294.5)	670.5 (929.7)	0.568
Eosinophil count, m (SD)	305.1 (195.9)	350.2 (238.1)	0.688	601.2 (798.1)	844.1 (932.1)	0.626
AER, m (SD)	3.2 (3.1)	0.2 (0.7)	0.016	3.0 (2.5)	1.3 (2.2)	0.217

ACT: asthma control test; AER: annual exacerbation rate; ARC: allergic rhinoconjunctivitis; FeNO: fractional exhaled nitric oxide; FEV1: forced expiratory volume in the first second; IgE: immunoglobulin; SD: standard deviation.

**Table 4:** Impact of dupilumab according to the presence of bronchiectasis after 12 months of treatment.

	Without Bronchiectasis (*n* = 13)			With Bronchiectasis (*n* = 7)		
	Basal	12 Months	*p*-Value	Basal	12 Months	*p*-Value
ACT, m (SD)	12.6 (4.1)	18.6 (4.8)	0.009	12.7 (6.6)	21.3 (4.6)	0.039
FEV1, m (SD)	70.3 (19.0)	74.0 (10.5)	0.347	68.4 (17.6)	53.5 (7.8)	0.851
FeNO, m (SD)	68.8 (47.8)	42.7 (37.4)	0.168	55.1 (47.6)	18.0 (7.0)	0.115
IgE, m (SD)	568.8 (445.3)	475.5 (320.4)	0.353	2211.7 (4597.3)	1094.5 (1342.8)	0.377
Eosinophils, m (SD)	315.4 (181.9)	400.0 (324.0)	0.487	601.4 (875.4)	973.3 (1253.8)	0.599
AER, m (SD)	3.8 (3.7)	1.1 (1.8)	0.032	2.8 (2.5)	0 (0)	0.025

ACT: asthma control test; AER: annual exacerbation rate; ARC: allergic rhinoconjunctivitis; FeNO: fractional exhaled nitric oxide; FEV1: forced expiratory volume in the first second; IgE: immunoglobulin; SD: standard deviation.

**Table 5:** Impact of dupilumab according to the presence of obesity after 12 months of treatment.

	Non-Obese (*n* = 20)			Obese (*n* = 8)		
	Basal	12 Months	*p*-Value	Basal	12 Months	*p*-Value
ACT, m (SD)	13.5 (5.5)	20.3 (4.5)	0.004	14.1 (5.6)	21.3 (5.5)	0.044
FEV1, m (SD)	75.9 (17.0)	74.6 (23.3)	0.555	68.5 (23.2)	70.7 (12.0)	0.442
FeNO, m (SD)	67.5 (58.9)	20.5 (8.1)	0.033	53.5 (22.5)	58.0 (56.6)	0.575
IgE, m (SD)	1280.4 (2734.5)	731.2 (798.2)	0.333	497.1 (382.2)	435.0 (323.4)	0.851
Eosinophils, m (SD)	385.5 (524.5)	736.7 (860.3)	0.228	400.0 (256.3)	400.0 (360.5)	1.000
AER, m (SD)	3.0 (3.4)	0.2 (0.7)	0.011	3.2 (1.3)	1.3 (2.1)	0.030

ACT: asthma control test; AER: annual exacerbation rate; ARC: allergic rhinoconjunctivitis; FeNO: fractional exhaled nitric oxide; FEV1: forced expiratory volume in the first second; IgE: immunoglobulin; SD: standard deviation.

**Table 6:** Impact of dupilumab according to the presence of allergic rhinoconjunctivitis after 12 months of treatment.

	Without Rhinoconjunctivitis (*n* = 10)			With Rhinoconjunctivitis (*n* = 18)		
	Basal	12 Months	*p*-Value	Basal	12 Months	*p*-Value
ACT, m (SD)	14.2 (5.9)	23.0 (2.7)	0.008	13.4 (5.2)	19.1 (5.0)	0.017
FEV1, m (SD)	73.2 (24.6)	92.0 (13.9)	0.543	74.0 (15.5)	61.8 (9.9)	0.942
FeNO, m (SD)	72.7 (53.9)	20.7 (5.8)	0.067	58.4 (50.4)	35.4 (30.6)	0.177
IgE, m (SD)	367.7 (244.0)	101.5 (61.5)	0.169	1439.4 (2856.2)	972.0 (732.41)	0.375
Eosinophils, m (SD)	471.0 (449.9)	1306.7 (965.4)	0.069	344.4 (175.6)	283.3 (213.7)	0.756
AER, m (SD)	2.7 (1.9)	0.5 (0.5)	0.012	3.3 (3.2)	0.8 (1.7)	0.024

ACT: asthma control test; AER: annual exacerbation rate; ARC: allergic rhinoconjunctivitis; FeNO: fractional exhaled nitric oxide; FEV1: forced expiratory volume in the first second; IgE: immunoglobulin; SD: standard deviation.

**Table 7:** Impact of dupilumab in the 15 patients switching from another biologic after 12 months of treatment.

	Patients with Previous Biologic Treatment (*n* = 15)		
	Basal	12 Months	*p*-Value
ACT, m (SD)	14.4 (5.5)	18.5 (4.7)	0.066
FEV1, m (SD)	70.1 (19.1)	74.0 (23.4)	0.713
FeNO, m (SD)	55.1 (51.6)	31.3 (33.1)	0.157
IgE, m (SD)	1463.1 (3122.5)	654.0 (806.0)	0.290
Eosinophil count, m (SD)	434.0 (597.3)	724.0 (976.7)	0.433
Annual exacerbation rate, m (SD)	3.8 (3.6)	1 (1.8)	0.015

ACT: asthma control test; AER: annual exacerbation rate; ARC: allergic rhinoconjunctivitis; FeNO: fractional exhaled nitric oxide; FEV1: forced expiratory volume in the first second; IgE: immunoglobulin; SD: standard deviation.

**Table 8:** Impact of dupilumab on nasal symptoms in patients with severe asthma and CRSwNP.

	Basal	12 Months	*p*-Value
SNOT22, m (SD)	78.3 (6.5)	38.3 (15.2)	0.013
NPS, m (SD)	2.1 (1.9)	0.6 (0.9)	0.134
TDI, m (SD)	7 (2.6)	18 (12.7)	0.214
Olfactory VAS, m (SD)	8.7 (3.4)	7.6 (2.5)	0.551
SVQOD, m (SD)	8.6 (5.7)	11 (7.1)	0.707
General VAS, m (SD)	8 (2.5)	4.2 (2.6)	0.028

NPS: nasal polyp score; SD: standard deviation; SNOT22: sinonasal outcome test (22 items); SVQOD: sinonasal quality of life questionnaire; TDI: threshold, discrimination, identification (the three subtests of the Sniffin’ sticks olfactory test); VAS: visual analog scale.

## Data Availability

The original contributions presented in this study are included in this article. Further inquiries can be directed to the corresponding author(s).
